# Dietary diversity, nutrition knowledge and attitude among adolescents in Addis Ababa: a cross-sectional study

**DOI:** 10.3389/fnut.2026.1696811

**Published:** 2026-04-07

**Authors:** Daniale T. Ekubagewargies, Patricia Lee, Faruk Ahmed

**Affiliations:** 1 Public Health, School of Medicine and Dentistry, Griffith University, Gold Coast, QLD, Australia; 2 Department of Paediatric and Child Health Nursing, School of Nursing, The University of Gondar, Gondar, Ethiopia

**Keywords:** adolescents, attitudes, dietary diversity, Ethiopia, knowledge, nutrition, urban health

## Abstract

**Background:**

Ethiopian urban adolescents are experiencing rapid dietary transitions that increase their risk of overweight and obesity, while undernutrition is still prevalent. Very few studies have explored their dietary practices or nutrition-related knowledge and attitudes. This study aimed to assess the dietary diversity, nutrition-related knowledge, and attitudes among school-going adolescents in Addis Ababa, Ethiopia.

**Methods:**

A school-based cross-sectional survey was conducted among 371 adolescents aged 14–19 years from public and private secondary schools using multi-stage cluster sampling. Dietary intake was assessed using a 24-h recall questionnaire. Structured questionnaires were used to collect data on nutrition knowledge and attitudes. Descriptive statistics and multivariate logistic regression were used to explore associations.

**Results:**

Only 33.7% of adolescents achieved adequate dietary diversity (≥5 food groups). While most participants consumed cereals daily, intake of fruits, vegetables, and animal-source foods was limited. The mean knowledge score was 11.27 (SD = 6.26) out of a possible 25 score. 51.2% of participants scored below the mean with major gaps in knowledge of micronutrient-rich food sources. Slightly more than half (52.7%) of participants had a favourable attitude using the mean score (60.6, SD = 7.47) as the cut-off. Private school attendance was associated with higher dietary diversity (AOR = 2.47, 95% CI: 1.50–4.08). Adolescents with secondary or tertiary educated fathers (AOR = 1.48, 95% CI: 1.07–2.04; AOR = 1.67, 95% CI: 1.16–2.39) and tertiary educated mothers (AOR = 1.82, 95% CI: 1.27–2.62) had better dietary diversity. In addition, higher nutrition knowledge scores were associated with higher odds of achieving adequate dietary diversity (AOR = 1.06, 95% CI: 1.01–1.10).

**Conclusion:**

Adolescents in Addis Ababa demonstrate suboptimal dietary diversity and limited nutrition knowledge, with pronounced socio-economic disparities. Urgent, school-based interventions that integrate nutrition education along with improvements to the food environment are essential to foster healthy dietary behaviours.

## Introduction

Adolescence is a pivotal life stage characterised by rapid growth and heightened nutritional demands (1). However, prevailing adolescent diets are often unbalanced, failing to meet these increased requirements. Such dietary inadequacies contribute to both nutrient deficiencies and excessive intake, thereby increasing the risk of undernutrition as well as overweight and obesity (2). In Low- and Middle-Income Countries (LMICs), adolescent malnutrition remains a pervasive challenge, manifesting as both undernutrition and emerging overnutrition. Pooled data from 57 LMICs indicate that, among adolescents aged 12–15 years, 10.2% experience stunting (low height-for-age) and 5.5% suffer from thinness (low BMI-for-age) (3). In East Africa, 16.5% of late adolescent girls (15–19 years) are undernourished (4). In Ethiopia, regional analyses reveal similar levels of adolescent (aged 12–15 years) stunting and thinness (5). A systematic review on the double burden of malnutrition among Ethiopian adolescents aged 10–19 years reported pooled prevalence rates of 10.63% for overweight/obesity, 20.06% for stunting, and 21.68% for thinness (6).

Malnutrition during adolescence can have lasting impacts on physical growth, cognitive development, and long-term health, including an increased risk of anaemia, adverse pregnancy outcomes, and heightened susceptibility to chronic disease (7, 8). Despite this global burden, adolescents have historically been underrepresented in nutrition policies and interventions, with much greater attention directed toward early childhood and maternal health (9).

While adolescence represents a critical period for establishing lifelong healthy eating habits (10), their dietary behaviours during this stage are often marked by poor dietary patterns. Common examples include increased consumption of processed and sugary foods, frequent meal skipping, particularly breakfast, and insufficient intake of fruits and vegetables (11). A 2024 multi-country study analysing data from adolescents aged 10–19 years across low-, middle and high-income countries found that dietary diversity was lowest in lower-middle-income countries, with adolescents consuming an average of only 3.5 out of 10 possible food groups per day. This limited dietary diversity was strongly associated with inadequate micronutrient intake among both boys and girls (12). Adolescents in LMICs often consume inadequately diversified diets, with both boys and girls showing low intake of fruits, vegetables, and other nutrient-rich foods (13). In Ethiopia, recent estimates indicate that only 39.24% of adolescents achieve adequate dietary diversity (14). Their diets are often monotonously based on grains or tubers, with limited intake of fruits, vegetables, and animal-source foods, resulting in widespread micronutrient deficiencies (14, 15).

In urban settings, dietary transitions and the proliferation of processed foods add a further dimension, as adolescents become “early adopters” of new food trends and are highly exposed to commercial food marketing (16). Consequently, many urban adolescents face a dual burden of malnutrition, characterised by persistent micronutrient deficiencies alongside increasing rates of overweight and diet-related non-communicable diseases (17). Adolescent dietary behaviours are shaped by a wide range of social, cultural, and environmental factors (9, 18). At the individual level, factors such as adolescents’ increasing autonomy over food choices, along with their knowledge, attitudes, and perceptions of healthy and unhealthy eating, play a crucial role in shaping their dietary behaviours (10, 19). Also, adolescents are influenced by peer norms and social dynamics in their food choices (10, 20). In addition, broader socio-economic and environmental determinants such as household income, access to diverse foods, and school food environments play a substantial role in shaping dietary behaviours and nutrient intake (12). Taken together, these individual, social, and structural influences underscore the multifaceted drivers of adolescent dietary practices in urban contexts.

International frameworks now increasingly recognise the critical importance of this life stage, with the World Health Organisation (WHO) and the Food and Agriculture Organisation of the United Nations (FAO) calling for strengthened action to improve adolescent diets and health. WHO’s global strategy for adolescent health and its 2018 guideline on adolescent nutrition advocate multi-sectoral interventions spanning micronutrient supplementation, nutrition education, and the creation of healthy school environments (9). In alignment with these global priorities, Ethiopia’s National Nutrition Program (21) and its National Adolescent and Youth Health Strategy (22) underscore the need to address both undernutrition and emerging diet-related risks among youth, in accordance with national targets for reducing stunting, anaemia, and obesity. Despite these policy commitments, significant gaps persist in understanding adolescent dietary behaviours, particularly in rapidly urbanising contexts where food environments and consumption patterns are evolving.

As mentioned earlier, socio-economic status (SES) plays a critical role in shaping adolescents’ access to diverse diets and health information. In Ethiopia, for instance, clear distinctions exist between public and private schools: public institutions predominantly serve students from lower-income households, while private schools enrol adolescents from relatively wealthier backgrounds who can afford higher tuition fees (23). These differences may translate into unequal access to nutritious foods and nutrition-related knowledge, placing adolescents from disadvantaged families at greater risk of dietary inadequacy (22, 23). Understanding how such socio-economic disparities interact with adolescents’ nutrition-related knowledge and attitudes is critical for designing equitable and context-specific interventions (18, 24). Although the role of schools in promoting adolescent nutrition is well established in both global and national frameworks (25), empirical data on the dietary practices, knowledge, and attitudes of urban adolescents in Ethiopia remain limited. Ethiopia’s formal education system includes secondary schooling (Grades 9–12), overseen by the Ministry of Education. Nutrition is not taught as a standalone compulsory subject at this level; instead, nutrition-related topics are introduced intermittently within broader subjects such as biology or physical education (26). Ethiopia also implements a national school feeding programme that reaches an estimated ~25% of schoolchildren. However, coverage is not universal, and secondary schools, especially in urban areas such as Addis Ababa, are less consistently included compared with primary schools (27). As Ethiopia’s capital, Addis Ababa provides an appropriate context to examine how urbanisation and socio-economic diversity shape adolescent nutrition. This study assessed the dietary practices, nutrition-related knowledge, and attitudes of school-going adolescents, and analysed how socio-economic status intersects with knowledge and attitude to influence dietary behaviour. In doing so, it addresses key evidence gaps on typical adolescent diets, micronutrient adequacy, and the role of knowledge and attitudes in shaping dietary intake.

## Materials and methods

### Study design

A cross-sectional survey was conducted from January 2024 to February 2024, in four selected high schools in Addis Ababa, Ethiopia. The target population included adolescents aged 14–19 from both private and public high schools within the city.

The research received ethical clearance from the Research Ethics Committee of Griffith University, Australia, under GU Ref no. 442/23.

### Sample size

To determine the required sample size for this study, we used the formula where: *Z* = 1.96 for a 95% confidence level, *p* = 0.323 (32.3% prevalence of adequate dietary practices from national data) (14) and *E* = 0.05 (margin of error),


$$n=(Z^{2*}\;p^{*}\;(1-p))/E^{2}$$

Substituting the values, initial sample size without adjustments n_0_ = (1.96^2^ × 0.323 × (1–0.323))/(0.05^2^) = 336.

Adjustment for design effect (DEFF = 1.2): n_1_ = 336 × 1.2 = 403.2.

Applying finite population correction (*N* = 1,800):


$$\begin{array}{c} n\_\mathrm{FPC}=403.2/(1+(403.2–1)/1800)\\ =403.2/(1+402.2/1800)\\ =403.2/1.2234\approx 329.6 \end{array}$$

And finally, adjusting for 20% non-response: *n*_final = 329.6 / (1–0.20) = 329.6/0.80 ≈ 412.

### Sampling technique

Multi-stage cluster sampling was applied to select participants. First, four schools were randomly selected from a city-wide list of public and private schools (*N* = 232), with two schools from each list. These schools were chosen for their representation of a socioeconomically diverse population. In Ethiopia, public education is free or comes with minimal cost. However, private education incurs a significant tuition fee. Thus, most of the students attending private schools come from a higher economic background (23). The selection of schools is expected to reflect the socio-economic discrepancy among the participants. Within each selected school, one section per grade (Grades 9–12) was randomly chosen. All students within the selected classrooms were considered for participation.

### Data collection

A letter of support was obtained from the local educational department to conduct the proposed study in the schools of Addis Ababa, Ethiopia. Additionally, permission was granted by the school principals to approach students within their respective schools. Students and parents were given an information letter and informed consent forms, and were asked to provide signed consent for participation. Meanwhile, students received a child-friendly version of the information sheet to help convey the information. After receiving the written consents from both the parents and adolescents, we included them in the study.

Data were collected by seven trained data collectors with backgrounds in public health and nursing, who received 2 days of training prior to data collection. The interviewer-administered questionnaire used in this study had four parts: SES, knowledge, attitude, and dietary intake. Socio-economic status was addressed with questions such as public vs. private school attendance and parental educational status.

#### Knowledge

Knowledge-related questions were also adapted from FAO (2014) guidelines for assessing nutrition-related Knowledge, Attitude, and Practice (KAP) (28) and other similar studies (29–31). There were 25 questions addressing participants’ knowledge on topics such as undernutrition, balanced diet, anaemia, vitamins and minerals, and obesity. All knowledge items allowed multiple responses and included a “do not know” option, consistent with FAO KAP survey design. During data entry and analysis, each item was scored dichotomously (0–1): a response was coded as correct (1) if the participant identified at least one correct option, and incorrect (0) if no correct option was identified or if “do not know” was selected. This yielded a total knowledge score ranging from 0 to 25. Because validated cut-offs for these adapted knowledge items are not established for Ethiopian adolescents, we used the sample mean as a pragmatic threshold to classify participants into lower versus higher knowledge, consistent with prior KAP applications in similar contexts. Scores below the sample mean indicated poor knowledge, while scores at or above the mean indicated good knowledge. In the analysis of knowledge questions, special adjustments were made for “Have you heard” gateway questions (e.g., have you heard about anaemia?). For these questions, subsequent related answers (e.g., causes, signs) were excluded if the participant responded “No” to the “Have you heard” question. This adjustment ensured that participants who indicated no prior awareness of a topic were not inadvertently credited for correct answers to follow-up questions.

#### Attitude

In the same manner, attitude-related questions were also adapted from the FAO (2014) guidelines for assessing nutrition-related KAP (28) and other similar studies (29–31). Sixteen attitude questions tap into areas of attitude such as perceived susceptibility, perceived severity, perceived barriers, and self-efficacy. These questions are presented in a 5-point Likert scale from 1 (e.g., strongly disagree) to 5 (strongly agree). Negative attitude questions were reverse-coded to ensure consistent interpretation and accurate representation of the data. For example, a response indicating strong disagreement with a negative statement was scored higher to reflect a positive attitude. This provides a minimum score of 16 and a maximum score of 80 per individual. In the absence of locally validated adolescent thresholds for this adapted attitude scale, the sample mean was used to classify favourable versus unfavourable attitudes to facilitate interpretability and comparison across subgroups. Scores below the mean indicated an unfavourable attitude, while scores at or above the mean indicated a favourable attitude.

#### Dietary practice

The dietary practice questionnaire was developed through adaptation of the FAO (2014) guidelines for assessing nutrition-related KAP (28) and FAO and Family Health International (FHI) 360 (2016) guidelines (32). The 24-h dietary recall, a structured interview questionnaire, was used to assess an individual’s food and beverage intake over the previous 24 h. It involves a detailed account of all foods and drinks consumed, including portion sizes, preparation methods, and brand names, providing a snapshot of dietary habits (33). To understand diet quality, dietary diversity score (DDS) was then calculated using *Minimum Dietary Diversity for Women: A Guide for Measurement* (FAO and FHI 360, 2016) (32). A participant is classified as having adequate DDS if they consumed foods from at least five of the 10 defined food groups within the past 24 h. The food groups are: Grains, white roots and tubers, and plantains; Pulses (beans, peas and lentils); Nuts and seeds; Dairy; Meat, poultry and fish; Eggs; Dark green leafy vegetables; Other vitamin A-rich fruits and vegetables; Other vegetables; and Other fruits. Although MDD-W was originally developed for women of reproductive age, we applied the same 10-food-group classification and ≥5 food-group threshold as a pragmatic, widely used indicator supported by recent multi-country evidence indicating that food-group diversity thresholds in this range meaningfully predict micronutrient adequacy among adolescents aged 10–19 years, supporting the interpretability of DDS-based metrics beyond women of reproductive age (12).

Data quality was ensured through pre-testing, regular supervision, and daily checks for completeness and consistency. The questionnaire was pilot-tested on 15 students from two schools that are not part of the current study. After this, the necessary adjustments were made to the content and presentation of the questionnaire for the final data collection.

Data collection was conducted during school hours, with the school principal’s permission allowing adolescents to be released from class to participate in the study.

### Data analysis

Data were analysed using IBM SPSS Statistics for Windows, Version 29.0 (IBM Corp., Armonk, NY). Descriptive statistics were used to summarise sample characteristics, including frequencies, means, and standard deviations for socio-demographic variables, nutrition knowledge, attitudes, and dietary diversity score (DDS).

Associations between dietary intake, socio-demographic factors, nutrition-related knowledge, and attitudes were examined using Chi-square tests of independence and independent-samples t-tests. Logistic regression models were used to assess associations between the primary outcome (DDS; low vs. high) and key independent variables. Variables were first examined in bivariate logistic regression, and those with *p* ≤ 0.25 were considered for inclusion in the multivariable model to avoid premature exclusion of potentially relevant predictors.

Secondary analyses modelled nutrition knowledge and attitude scores (each dichotomised) as outcomes. Specifically, we examined: (i) the effects of socio-economic status indicators (e.g., school type, parental education and occupation); (ii) the associations of these factors with knowledge and attitudes; and (iii) whether knowledge and attitudes were independently associated with dietary practices. Collinearity was assessed using Variance Inflation Factors (VIF) and tolerance values, with VIF > 5 or tolerance < 0.2 indicating potential multicollinearity.

A *p*-value < 0.05 was considered statistically significant. Results are presented as means, proportions, odds ratios, and 95% confidence intervals.

## Results

From the selected classrooms, there were 393 students. Out of the 393 questionnaires distributed, 371 were complete, providing a response rate of 94.4%. One hundred ninety-three (52%) of the participants were from public schools. Regarding gender representation, 53.6% were female. Grade 9 & 10 students constitute the majority, contributing 27.8 and 26.1% of the participants. The mean age of students was 16.65 years (SD = 1.39). Regarding parental education level, 45.6% of fathers and 35.3% of mothers have a tertiary-level education (Table 1).

**Table 1:** Socio-demographic characteristics of adolescents attending secondary schools in Addis Ababa, Ethiopia (*N* = 371).

Variable	Category	*N*	%
Type of school	Public	193	52.0
	Private	178	48.0
Age group	Mid-Adolescent (14–17)	266	71.7
	Late-Adolescent (18–19)	105	28.3
Grade	Grade 9	103	27.8
	Grade 10	97	26.1
	Grade 11	75	20.2
	Grade 12	96	25.9
Gender	Male	172	46.4
	Female	199	53.6
Parents living together	Yes	277	74.7
	No	94	25.3
Father’s education	No formal education	28	7.5
	Primary	58	15.6
	Secondary	116	31.3
	Tertiary	169	45.6
Mother’s education	No formal education	50	13.5
	Primary	73	19.7
	Secondary	117	31.5
	Tertiary	131	35.3
Father’s occupation	Government employee	76	20.5
	Business owner	222	59.8
	Farmer	19	5.1
	Unemployed	54	14.6
Mother’s occupation	Government employee	64	17.3
	Business owner	161	43.4
	Farmer	25	6.7
	Unemployed	121	32.6
Household size	Small (5 or less people)	221	59.6
	Large (more than 5 people)	150	40.4

### Dietary practice

**Figure 1:**
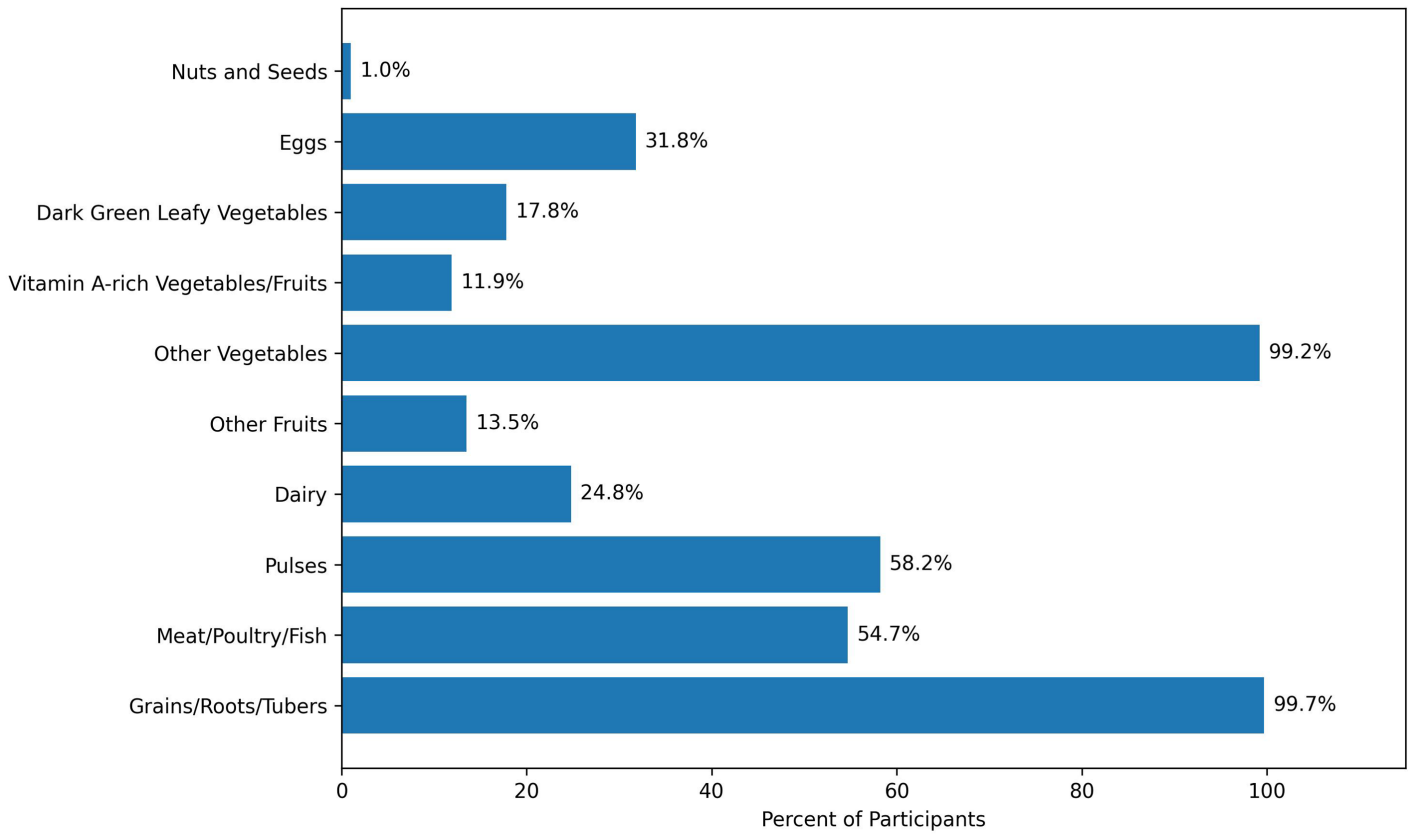
Percentage of adolescents consuming at least one item from each of the ten food groups in the previous 24 hours, Addis Ababa (*n* = 371).

Regarding food group consumption within the past 24 h, nearly all participants (99.7%) consumed grains, white roots, tubers, and plantains (Figure 1, Percentage of Participants Consuming Each Food Group in 24 h (*n* = 371)). However, the consumption of nutrient-dense foods such as nuts and seeds (3.0%), dark green leafy vegetables (17.8%), and other vitamin A-rich fruits and vegetables (11.9%) was very low. Similarly, dairy products were consumed by only 24.8% of participants, while 54.7% consumed meat, poultry, or fish, and 31.8% consumed eggs. Additionally, 67.4% of participants reported consuming sweets or sugar-sweetened beverages within the past 24 h.

### Dietary diversity score

**Figure 2:**
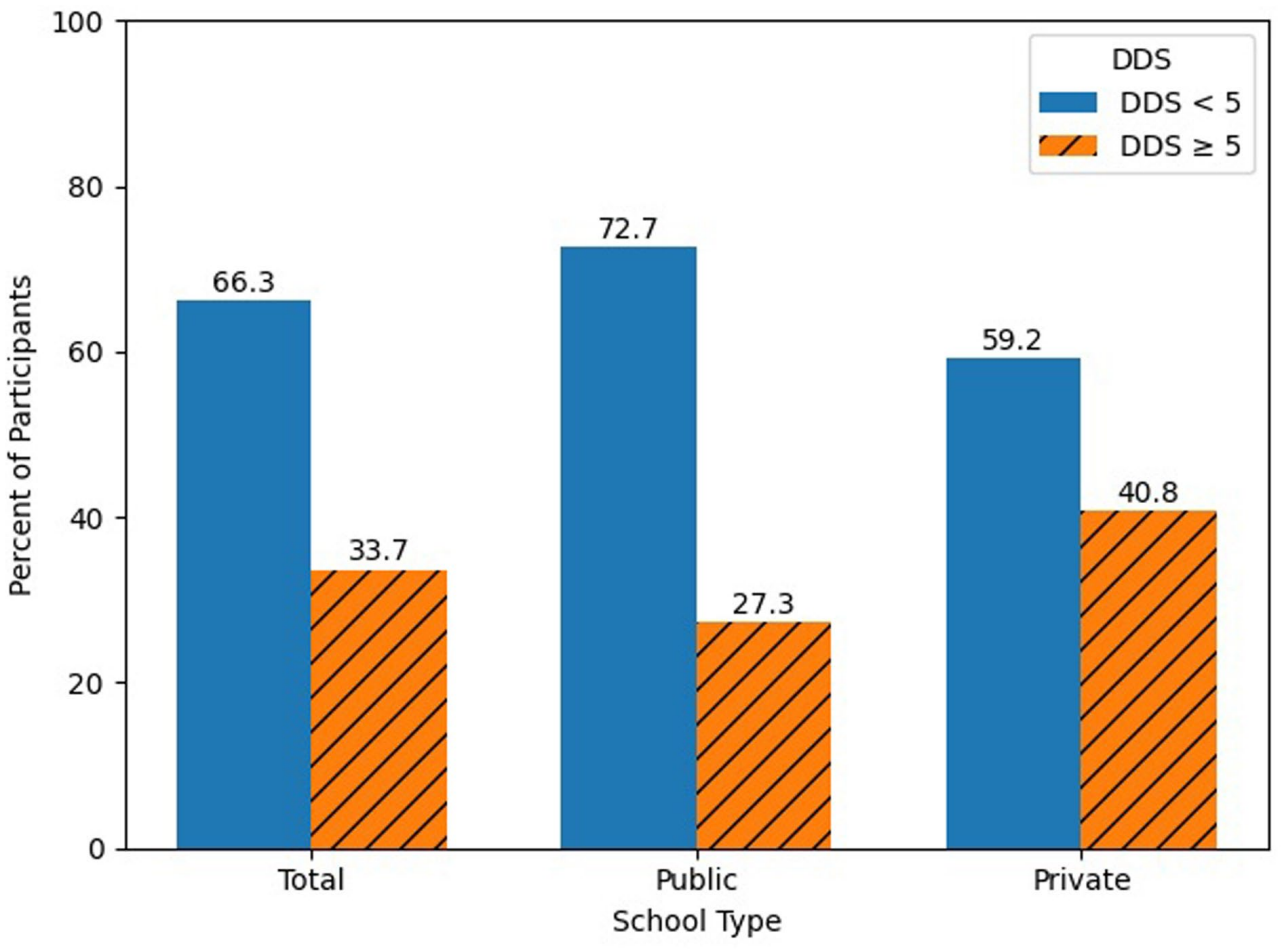
Proportion of Participants with Adequate and Inadequate Dietary Diversity Score Per School Type (*n* = 371).

Based on the 24-h food recall, the mean DDS was 4.2, highlighting that, on average, adolescents consumed foods from four food groups, out of ten, in the past 24 h. Overall, only 33.7% of participants achieved a DDS of five or more food groups. More students from private schools (48.3%) had adequate DDS compared to public school students (20.2%). Figure 2, Proportion of Participants with Adequate and Inadequate DDS Per School Type (*n* = 371).

### Nutrition knowledge

Overall, participants demonstrated moderate knowledge of dietary and nutritional topics with a mean knowledge score of 11.27 (SD = 6.26) and scores following a normal distribution. Only 48.8% of participants had a good knowledge, defined as scoring at or above the mean. Awareness of specific topics varied widely. While 47.4% recognised the causes of anaemia, only 30.2% identified iron-rich foods, and just 8.9% were aware of food sources of vitamin D. In contrast, 41.8% could name iodine-rich foods, 25.1% were familiar with calcium sources, and 34.8% identified sources of vitamin A. General nutritional awareness was higher, with 75.2% knowledgeable about high-fat foods and 71% aware of obesity prevention strategies (See Table 2). Female students were significantly more likely than males to have high nutrition knowledge (AOR = 2.62; 95% CI: 1.62–4.22). Students in Grade 10 (AOR = 1.51; 95% CI: 1.17–1.96) and Grade 11 (AOR = 2.78; 95% CI: 1.22–6.36) were also more likely to demonstrate high knowledge compared to those in Grade 9. Attending a private school was strongly associated with greater knowledge (AOR = 2.79; 95% CI: 1.64–4.73). In contrast, adolescents whose mothers were farmers were significantly less likely to have high knowledge (AOR = 0.10; 95% CI: 0.02–0.57) (Table 3).

**Table 2:** Knowledge of nutrition-related topics among adolescents in secondary schools in Addis Ababa, Ethiopia (*N* = 371).

Knowledge item	Total correct (*n*)	Total correct (%)	Boys (%)	Girls (%)	*p*-value
Foods high in fat content	279	75.2	65.1	83.9	<0.001
Balanced diet purpose	273	73.6	66.9	79.4	0.006
Problems associated with overweight and obesity	268	72.2	65.1	78.4	0.004
Prevention of overweight/obesity	263	70.9	70.9	70.9	0.987
Signs of malnutrition	231	62.3	53.5	69.8	0.001
Consequences of not eating breakfast	226	60.9	51.7	68.8	<0.001
Signs of anaemia	199	53.6	43.0	62.8	<0.001
Reasons for overweight/obesity	187	50.4	45.3	54.8	0.070
Benefits of vitamins and minerals	181	48.8	43.6	53.3	0.063
Balanced diet definition	177	47.7	39.5	54.8	0.003
Causes of anaemia	176	47.4	36.0	57.3	<0.001
Iodine deficiency signs	171	46.1	39.0	52.3	0.010
Iodine deficiency causes	171	46.1	39.0	52.3	0.010
Vitamin D deficiency signs	167	45.0	41.3	48.2	0.179
Vitamin D deficiency causes	167	45.0	41.3	48.2	0.179
Iodine-rich foods	155	41.8	34.9	47.7	0.012
Vitamin A deficiency signs	133	35.8	29.1	41.7	0.011
Foods rich in vitamin A	129	34.8	29.1	39.7	0.032
Calcium deficiency signs	124	33.4	30.8	35.7	0.322
Iron-rich foods	112	30.2	26.7	33.2	0.179
Vitamin A deficiency causes	97	26.1	22.7	29.1	0.157
Calcium deficiency causes	93	25.1	22.7	27.1	0.323
Calcium-rich foods	93	25.1	22.7	27.1	0.323
Reasons for undernutrition	78	21.0	14.0	27.1	0.002
Vitamin D–rich foods	33	8.9	7.0	10.6	0.228

Values represent the proportion of adolescents providing at least one correct response per item. Percentages for boys and girls are calculated within sex. *p*-values are from Pearson’s chi-square tests. (*N* = 371; boys *n* = 172, girls *n* = 199).

**Table 3:** Multivariable logistic regression of factors associated with nutrition knowledge among adolescents (*N* = 371).

Predictor variable		AOR	95% CI for AOR	*p*-value
Gender	Male (Ref)	1.00	–	–
	Female	2.47	1.56–3.92	<0.001
Type of school:	Public (Ref)	1.00	–	–
	Private	2.84	1.72–4.69	<0.001
Grade	Grade 9 (Ref)	1.00	–	–
	Grade 10	1.51	1.17–1.96	0.002
	Grade 11	2.78	1.22–6.36	0.001
	Grade 12	1.27	0.67–2.40	0.073
Maternal occupation	Gov’t Employee (Ref)	1.00	–	–
	Business owner	0.94	0.72–1.23	0.659
	Farmer	0.67	0.02–0.57	0.10
	Unemployed/other	0.78	0.33–1.83	0.756

Adjusted for gender, type of school, grade, and maternal occupation.

### Attitudes toward healthy dietary behaviour

Participants’ attitudes toward healthy dietary behaviour demonstrated variability, with scores ranging from 35.00 to 80.00 and a mean of 60.6 (SD = 7.47). Slightly more than half (52.7%) of adolescents were classified as having a favourable attitude toward healthy dietary practices. The majority of adolescents expressed strong beliefs in the importance of nutritious food (70.9%) and breakfast (52.0%), fewer reported consistently following a healthy diet (only 11.6% highly likely). In addition, although many recognised the seriousness of undernutrition (62.0%) and obesity (69.3%), their food choices were often shaped by cost (48.5% prioritised cost over healthiness) and personal preference (25.6% ate what they liked regardless of health) (Table 4). Gender and father’s occupation were significant predictors of adolescents’ favourable attitudes toward healthy dietary behaviour. Specifically, female students had nearly twice the odds of reporting a high attitude score compared to their male counterparts (AOR = 1.93; 95% CI: 1.23–3.02), indicating a gendered difference in nutrition-related attitudes. Furthermore, adolescents whose fathers were farmers had significantly greater odds of holding favourable attitudes (AOR = 4.91; 95% CI: 1.15–20.99) compared to those whose fathers were government employees (Table 5).

**Table 4:** Attitude toward dietary behaviour among adolescents in secondary schools in Addis Ababa, Ethiopia (*N* = 371).

Question	Scale answer				
	1 (*n*, %)	2 (*n*, %)	3 (*n*, %)	4 (*n*, %)	5 (*n*, %)
1. The food I eat must be nutritious.	7 (1.9)	4 (1.1)	26 (7.0)	71 (19.1)	263 (70.9)
2. I always follow a healthy and balanced diet.	31 (8.4)	47 (12.7)	150 (40.4)	100 (27.0)	43 (11.6)
3. I eat what I like and do not worry about healthiness.	51 (13.7)	44 (11.9)	87 (23.5)	76 (20.5)	113 (30.5)
4. I consider cost over healthiness when buying food.	117 (31.5)	63 (17.0)	57 (15.4)	49 (13.2)	84 (22.6)
5. The healthiness of food has little impact on my choices.	141 (38.0)	60 (16.2)	62 (16.7)	49 (13.2)	59 (15.9)
6. How serious is undernutrition for adolescents?	21 (5.7)	11 (3.0)	44 (11.9)	65 (17.5)	230 (62.0)
7. How committed are you to healthy eating to prevent it?	21 (5.7)	27 (7.3)	75 (20.2)	99 (26.7)	145 (39.1)
8. How serious is vitamin deficiency?	20 (5.4)	20 (5.4)	53 (14.3)	93 (25.1)	185 (49.9)
9. How important is eating breakfast?	8 (2.2)	21 (5.7)	57 (15.4)	89 (24.0)	193 (52.0)
10. How serious are complications from obesity?	21 (5.7)	16 (4.3)	30 (8.1)	47 (12.7)	257 (69.3)
11. How committed are you prevent yourself from obesity?	26 (7.0)	12 (3.2)	59 (15.9)	87 (23.5)	186 (50.1)
12. How important is physical activity?	10 (2.7)	9 (2.4)	21 (5.7)	57 (15.4)	273 (73.6)
13. How easy is doing physical activity for you?	44 (11.9)	43 (11.6)	78 (21.0)	95 (25.6)	110 (29.6)
14. Are you satisfied with the nutrition knowledge you have?	34 (9.2)	30 (8.1)	110 (29.6)	107 (28.8)	88 (23.7)
15. How willing are you to get nutrition education?	22 (5.9)	23 (6.2)	62 (16.7)	72 (19.4)	188 (50.7)
16. How willing are you to learn from school media?	21 (5.7)	18 (4.9)	79 (21.3)	78 (21.0)	174 (46.9)

Attitudes toward nutrition and healthy eating were assessed using a five-point Likert scale ranging from 1 = strongly disagree to 5 = strongly agree. Negatively worded items were reverse-coded prior to analysis so that higher scores consistently reflected more favourable attitudes.

**Table 5:** Multivariable logistic regression of factors associated with nutrition attitudes among adolescents (*N* = 371).

Predictor variable		AOR	95% CI for AOR	*p*-value
Gender	Male (Ref)	1.00	–	–
	Female	1.93	1.23–3.02	0.004
Father Occupation	Gov’t employee (Ref)	1.00	–	–
	Business owner	0.60	0.32–1.13	0.110
	Farmer	4.91	1.15–20.99	0.031
	Unemployed/other	0.78	0.33–1.83	0.756

Adjusted for gender and father’s occupation.

### Bivariate analysis with DDS

The bivariate analysis showed that adolescents attending private schools had a significantly higher proportion achieving DDS ≥ 5 compared with those in public schools (48.3% vs. 20.2%, *p* < 0.001). DDS also differed significantly across levels of parental education, with overall differences observed for both father’s education (*p* < 0.001) and mother’s education (*p* = 0.003). In addition, nutrition knowledge was significantly associated with DDS, with a higher proportion of adolescents with high knowledge scores achieving DDS ≥ 5 compared with those with lower knowledge scores (42.0% vs. 25.8%, *p* < 0.001) (Table 6).

**Table 6:** Bivariate associations between selected variables and dietary diversity score (*N* = 371).

Variable	Category	DDS < 5, *n* (%)	DDS ≥ 5, *n* (%)	*p*-value
Type of school				<0.001
	Public	154 (79.8)	39 (20.2)	
	Private	92 (51.7)	86 (48.3)	
Gender				0.080
	Male	122 (70.9)	50 (29.1)	
	Female	124 (62.3)	75 (37.7)	
Age group				0.692
	Mid-adolescence (14–17)	178 (66.9)	88 (33.1)	
	Late-adolescence (18–19)	68 (64.8)	37 (35.2)	
Grade				0.290
	Grade 9	69 (67.0)	34 (33.0)	
	Grade 10	71 (73.2)	26 (26.8)	
	Grade 11	45 (60.0)	30 (40.0)	
	Grade 12	61 (63.5)	35 (36.5)	
Parents living together				0.354
	Yes	180 (65.0)	97 (35.0)	
	No	66 (70.2)	28 (29.8)	
Household size				0.582
	≤5 people	149 (67.4)	72 (32.6)	
	>5 people	97 (64.7)	53 (35.3)	
Father’s education				<0.001
	No formal education	22 (78.6)	6 (21.4)	
	Primary	46 (79.3)	12 (20.7)	
	Secondary	87 (75.0)	29 (25.0)	
	Tertiary	91 (53.8)	78 (46.2)	
Mother’s education				0.003
	No formal education	38 (76.0)	12 (24.0)	
	Primary	57 (78.1)	16 (21.9)	
	Secondary	79 (67.5)	38 (32.5)	
	Tertiary	72 (55.0)	59 (45.0)	
Father’s occupation				0.450
	Government employee	45 (59.2)	31 (40.8)	
	Business owner	149 (67.1)	58 (32.9)	
	Farmer	14 (73.7)	4 (26.3)	
	Unemployed/other	38 (70.4)	12 (29.6)	
Mother’s occupation				0.895
	Government employee	45 (70.3)	19 (29.7)	
	Business owner	105 (71.9)	41 (28.1)	
	Farmer	16 (84.2)	3 (15.8)	
	Unemployed/other	80 (66.1)	41 (33.9)	
Nutrition knowledge				<0.001
	Low (≤11)	141 (74.2)	49 (25.8)	
Attitude	High (>11)	105 (58.0)	76 (42.0)	
				0.875

Values are presented as *n* (%), with percentages calculated within each category. Associations between categorical variables and dietary diversity score (DDS) were assessed using Pearson’s chi-square test. For variables with more than two categories, *p*-values reflect overall group differences.

### Regression analysis

Based on regression analysis, Type of School, Gender, Father’s Education, Mother’s Education, and Knowledge were selected. In addition, Attitude was included in the model based on theoretical relevance, despite not meeting the statistical threshold in the bivariate analysis. All selected variables were entered simultaneously into the initial multivariable logistic regression model, from which the final model was derived. The final model is shown in Table 7. Overall, attending a private school was significantly associated with higher odds of achieving a high DDS compared to attending a public school (AOR = 2.469, 95% CI: 1.50–4.08, *p* < 0.001). Parental education was also significantly associated with dietary diversity. Adolescents whose fathers had secondary or tertiary education had higher odds of attaining a high DDS compared to those whose fathers had no formal education (secondary: AOR = 1.48, 95% CI: 1.07–2.04, *p* = 0.022; tertiary: AOR = 1.67, 95% CI: 1.16–2.39, *p* = 0.006). Similarly, maternal tertiary education was a significant predictor (AOR = 1.82, 95% CI: 1.27–2.62, *p* = 0.002), while primary and secondary education reached marginal significance. Participants with good knowledge had higher odds of achieving a high DDS compared to those with poor knowledge (AOR = 1.058, 95% CI: 1.01–1.10, *p* = 0.009). Attitude was not a significant predictor in the final model (*p* = 0.424). The Hosmer–Lemeshow test indicated a good model fit (*p* = 0.961).

**Table 7:** Multivariable logistic regression analysis of dietary diversity score (*N* = 371).

Predictor	*B*	SE	Wald	df	*p*-value	AOR	95% CI
Type of school							
Public (ref)							
Private	0.904	0.266	11.536	1	<0.001	2.469	1.50–4.08
Father’s education							
No formal education (ref)							
Primary	0.203	0.151	1.808	1	0.179	1.22	0.91–1.65
Secondary	0.39	0.17	5.268	1	0.022	1.48	1.07–2.04
Tertiary	0.51	0.185	7.598	1	0.006	1.67	1.16–2.39
Mother’s education							
No formal education (ref)							
Primary	0.29	0.169	2.948	1	0.086	1.34	0.96–1.86
Secondary	0.32	0.175	3.343	1	0.068	1.38	0.98–1.94
Tertiary	0.6	0.19	9.975	1	0.002	1.82	1.27–2.62
Knowledge							
Poor knowledge (ref)							
Good knowledge	0.056	0.021	6.915	1	0.009	1.058	1.01–1.10
Attitude							
Unfavourable attitude (ref)							
Favourable attitude	0.012	0.015	0.64	1	0.424	1.012	0.98–1.04

Adjusted for type of school, gender, father’s education, mother’s education, nutrition knowledge score, and attitude score.

## Discussion

This quantitative study on adolescents in Addis Ababa provides valuable insights into dietary practices, nutrition-related knowledge, and attitudes. By including students from both public and private schools, it offers a broader understanding of adolescent dietary behaviour across diverse socio-economic settings.

Overall, dietary diversity was suboptimal among a substantial proportion of the participants. Using a 24-h recall, nearly two-thirds of the students did not meet the minimum recommended intake of five food groups per day; in our sample, only about 34% of adolescents achieved an adequate DDS. This finding aligns with prior reports from Ethiopia, where the pooled prevalence of adequate dietary diversity among adolescents is approximately 39% (14). The diets of these urban adolescents were predominantly based on staple grains, with comparatively lower consumption of fruits, vegetables, and animal-source foods – a pattern consistent with national surveys showing that monotonous diets contribute to widespread micronutrient gaps (14, 34).

While most students consumed grains daily, fewer consumed nutrient-rich foods like dairy, eggs, or green vegetables regularly. This imbalance raises concerns about micronutrient deficiencies; indeed, national data show high rates of anaemia and vitamin deficiencies among Ethiopian adolescents (5). Inadequate consumption of nutrient-rich foods like dairy, eggs, and green vegetables among adolescents increases the risk of micronutrient deficiencies, particularly calcium, iron, and vitamin A, which are linked to impaired growth, weakened immunity, and poor cognitive performance (7).

The adolescents’ nutrition-related knowledge and attitudes varied widely, revealing critical gaps. The mean nutrition knowledge score was 11.27 (SD = 6.26) out of 25, indicating moderate knowledge at best. Students demonstrated a reasonably good understanding of basic nutrition concepts, with nearly half able to define or explain the purpose of a balanced diet. However, their knowledge of specific micronutrients was limited. For instance, fewer than one-third of respondents could identify foods rich in iron or vitamin A, and an even smaller proportion (8.9%) were aware of key sources of vitamin D. These knowledge gaps are concerning and align with findings from other regions of Ethiopia, where adolescents often exhibit limited awareness of micronutrients and healthy diet guidelines (7, 34). The limited awareness of micronutrients among adolescents represents a significant public health concern, as it undermines their ability to make balanced food choices essential for growth, cognitive development, and long-term health (35, 36). Addressing these knowledge gaps requires targeted nutrition education interventions designed to improve awareness and promote healthier behaviours (36).

Attitudes toward healthy eating were generally favourable. The mean attitude score was 61.02 (SD = 7.47), out of a total score 80, with 52.7% of students having a favourable attitude. A large majority agreed that food should be nutritious and acknowledged the benefits of good eating habits. Nevertheless, a subset of adolescents expressed less favourable attitudes or expressed perceived barriers to healthy eating (for example, some were not convinced of personal susceptibility to nutrition-related health issues, or felt healthy foods are not easily accessible). While many adolescents valued the benefits of nutritious food and recognised the seriousness of both undernutrition and obesity, fewer consistently followed healthy diets, with cost and preference often taking precedence over health considerations. These findings suggest that practical or motivational challenges play a significant role in limiting the adaptation of healthy eating behaviours.

### Association between nutrition knowledge, attitudes, and DDS

Our study found that nutrition-related knowledge was a significant predictor of DDS among adolescents. Adolescents who were knowledgeable about were 5.8% more likely to have a diverse diet (AOR = 1.058, 95% CI: 1.01–1.10, *p* = 0.009), emphasising the role of cognitive understanding in shaping dietary choices. This finding aligns with previous studies showing that better nutrition knowledge is associated with healthier food choices and increased intake of fruits and vegetables (37, 38). This is supported by behavioural change theory, which emphasises that accurate knowledge is a foundational enabler of health-related behaviours when other factors like motivation or access are limited (37).

Although more than half of the adolescents expressed favourable attitudes toward healthy eating, attitude was not independently associated with DDS in multivariable analysis. This apparent disconnect highlights the distinction between motivational factors and actual dietary behaviour. While positive attitudes reflect awareness and intention, they may be insufficient to influence food choices in the absence of enabling conditions. Similar gaps between attitude and behaviour have been reported in previous adolescent nutrition studies, where environmental and social barriers weaken the influence of attitudes on dietary practices (39, 40). In contrast, nutrition knowledge, a cognitive factor, remained significantly associated with DDS, suggesting that understanding of nutrition may play a more direct role in guiding food selection when options are constrained. Consistent with the Information–Motivation–Behavioural Skills (IMB) model, motivation must be accompanied by adequate knowledge and practical opportunities for behaviour change to occur (41). Structural constraints, including socio-economic disadvantage, food affordability, limited access to diverse foods, and peer norms, are therefore likely to hinder adolescents’ ability to act on positive intentions (42, 43). These findings underscore that interventions focusing solely on attitude change are unlikely to be effective unless accompanied by strategies that improve nutrition knowledge and address environmental and economic barriers within school and household food systems.

### Socio-economic disparities in dietary practices, knowledge and attitude

A key finding of this study is the pronounced influence of socio-economic factors – captured primarily by school type (public vs. private used a proxy of SES) and parental education – on adolescents’ nutrition knowledge, attitude and dietary intake. Students attending private schools had significantly higher DDS and slightly better nutrition knowledge than those in public schools. In our sample, nearly half of private school students (48.3%) achieved a high DDS, compared to only around one-fifth (20.2%) of public school students (the latter often coming from less-educated or lower-income households). Multivariate analysis confirmed that attending a private school was associated with more than double the odds of having an adequately diverse diet (AOR = 2.469, 95% CI: 1.50–4.08, *p* < 0.001). This disparity aligns with the broader literature showing that higher SES is associated with better diet quality among adolescents (18, 24). Wealthier families can afford a wider variety of foods, such as protein-rich foods, fruits, and fortified products, and are less constrained by food insecurity. In addition, higher SES is often linked to greater exposure to nutrition information and healthier food environments. For example, a systematic review among adolescents in high-income countries found that healthier dietary patterns such as higher DDS, increased intake of fruits, vegetables, and dairy were consistently associated with higher parental SES, particularly higher levels of parental education (24). In contrast, families with limited resources may struggle to afford a variety of nutritious foods, leading to a consumption of less diverse foods (44). These findings underscore the need to address economic and educational inequalities as part of strategies to improve adolescents’ nutritional outcomes.

Adolescents whose fathers had attained secondary education were 1.48 times more likely to have a higher DDS compared to those whose fathers had no formal education (AOR = 1.48, 95% CI: 1.07–2.04, *p* = 0.022). This likelihood increased further among those with tertiary-educated fathers, who had 1.67 times higher odds of achieving high DDS (AOR = 1.67, 95% CI: 1.16–2.39, *p* = 0.006). Maternal education showed a similar pattern: adolescents whose mothers had a tertiary education were 1.82 times more likely to meet the minimum dietary diversity threshold than those whose mothers had no formal education (AOR = 1.82, 95% CI: 1.27–2.62, *p* = 0.002). Studies in Ethiopia and other settings have found similar findings where adolescents with more educated parents (particularly mothers) are significantly more likely to achieve minimum dietary diversity (34, 45). These findings illustrate how higher parental education can influence adolescents’ dietary behaviours by improving household nutrition literacy and better availability of food options. In one review conducted in Ethiopia, the odds for adolescents achieving minimum dietary diversity was 4.49 (95% CI: 2.14–9.41) when mothers had formal education, compared to those whose mothers had no formal education (34). Also, parents with greater awareness of healthy eating may transmit this knowledge to their children, enabling adolescents to more actively monitor and guide their own diet.

It is noteworthy that we observed very limited differences in DDS between male and female students. Unlike some studies in other Ethiopian settings that found girls to have poorer dietary intake or higher malnutrition risk than boys (39), our urban cohort showed comparable dietary patterns for both sexes. This could suggest that in an urban environment, gender-based disparities in diet are less pronounced, or that both boys and girls in Addis Ababa are equally exposed to nutrition transitions (including both healthy and unhealthy food options).

This study identified several important socio-demographic determinants of adolescents’ nutrition knowledge and attitudes in an urban Ethiopian context. For example, private school students were nearly three times more likely to exhibit high knowledge than public school students. This disparity likely reflects broader socioeconomic differences between public and private school populations, including differences in curricular emphasis, access to learning resources, and possibly parental educational background. Private school students may also have greater access to informal nutrition information through the media (46), extracurricular learning, or home environments where nutrition is prioritised.

Female students were noticeably more likely than males to have high levels of nutrition knowledge, indicating possible gender-related differences in exposure or interest in nutrition content. This finding supports previous research in Ethiopia (47) and Kenya (48), Australia (49) and Iran (50). This could be that girls show greater engagement with health and nutrition topics, possibly due to traditional gender roles that socialise them to be more health-conscious or responsible for household food practices.

Grade level was also positively associated with knowledge, with students in Grades 10 and 11 showing significantly higher odds of scoring well compared to those in Grade 9. This may reflect increased academic exposure to health and science curricula as students progress through secondary school.

Among parental SES indicators, maternal occupation remained independently associated with nutrition knowledge after adjustment. Adolescents whose mothers were farmers were less likely to possess high knowledge scores. Maternal employment in low-skilled and informal sectors, including agriculture, is often associated with limited access to health and nutrition information, particularly when combined with low maternal education (51). Farming, as a form of informal and time-intensive work, may reduce opportunities for mothers to engage with school-based nutrition messaging or community health initiatives. This finding may reflect underlying disparities in health literacy and access to nutrition information among households engaged in agriculture, particularly where mothers have limited formal education or minimal exposure to structured nutrition education platforms. It may also point to broader structural disadvantages commonly experienced by farming families in urban or peri-urban areas, such as lower household income or reduced interaction with school-based or community-driven nutrition literacy initiatives.

In terms of attitudinal outcomes, the analysis again revealed a significant gender effect: female adolescents were nearly twice as likely as males to report favourable attitudes toward nutrition. This supports the notion that gender differences not only affect knowledge acquisition but also influence how adolescents internalise and prioritise healthy dietary behaviours. Given that attitudes are critical precursors to behavioural change, this gender difference stresses the need for targeted interventions that better engage male students in nutrition discourse (52).

Paternal occupation, but not maternal occupation, was independently associated with adolescents’ nutrition attitudes; somewhat unexpectedly, adolescents whose fathers were farmers reported more favourable attitudes than those whose fathers were government employees, a pattern also noted in previous studies. A study among high school students revealed that adolescents with direct agricultural experience often demonstrate greater openness, willingness to try, and positive attitudes toward fruits and vegetables compared to peers without such exposure (53). This may be because growing up in farming households familiarises youth with fresh produce and promotes appreciation for food quality and self-sufficiency. These findings suggest that cultural norms in farming communities, including respect for homegrown foods and traditional preparation methods, may foster more favourable nutrition attitudes despite socioeconomic disadvantages relative to government employed households.

Together, these findings point to the complex interplay between gender, educational exposure, parental roles, and school type in shaping adolescents’ nutrition knowledge and attitudes. The divergence between knowledge and attitude predictors in the reversed pattern in parental occupation suggests that different mechanisms influence information access and attitudinal orientation. Interventions should therefore consider tailored strategies: enhancing access to accurate nutrition knowledge for underserved groups (e.g., public school students, children of farming mothers) while fostering more inclusive, gender-sensitive communication that resonates with both boys and girls.

### Strengths and limitations

This study has several strengths. To our knowledge, it is one of the few that focus on urban adolescent nutrition KAP in Ethiopia, a demographic often overlooked in nutrition research. The study employed a relatively large sample drawn from multiple schools, including both public and private institutions, enabling insights across varying socio-economic and educational settings. This broad approach supports a more comprehensive understanding of adolescent nutrition in similar urban environments. We used standardised and validated instruments to assess knowledge, attitudes, and dietary practices, adopting reputable guidelines from FAO and other similar studies. The data collection was carried out rigorously by trained interviewers, and the analysis accounted for key confounders, lending credibility to the associations observed (for example, adjusting for parental education when examining school-type differences). Moreover, by examining knowledge, attitudes, and practices together, our study offers a complete picture that can inform multifaceted interventions. Nonetheless, certain limitations must be acknowledged. First, the cross-sectional design restricts our ability to infer causality. For example, we did not conduct formal sensitivity analyses using alternative cut-offs (e.g., tertiles/quartiles or externally defined thresholds); therefore, the robustness of dichotomised classifications should be interpreted cautiously. The second limitation is that we cannot be certain that better knowledge leads to improved dietary diversity, only that they are related. Given the cross-sectional design, directionality cannot be established and reverse causality is plausible; for example, adolescents consuming more diverse diets may also be more exposed to nutrition information and thus score higher on knowledge assessments. Longitudinal or interventional studies would be necessary to determine the direction of these relationships. Third, dietary intake was assessed using a single 24-h recall, which captures recent consumption but does not necessarily represent usual intake due to within-person day-to-day variation, weekday–weekend differences, and seasonality. As a result, DDS may be under- or over-estimated for some participants depending on whether the recall day was atypical. Repeated recalls or usual-intake modelling would provide a more stable estimate of habitual dietary diversity. SES is multidimensional, and we did not collect direct household income or asset-based indices; therefore, SES may not have been fully captured, which may limit precision and allow residual confounding in observed associations with DDS, knowledge, and attitudes. The study relied on self-reported dietary data, which are prone to recall bias and social desirability bias. Participants might not accurately remember all foods consumed or may under-report unhealthy foods and over-report healthy ones. However, we sought to reduce this risk by using trained interviewers and cross-checking portion sizes with examples. Third, although our sample included both public and private schools, it was limited to high-school students in the capital city; therefore, the findings may not apply to out-of-school adolescents or those in rural areas.

In conclusion, this study emphasises both challenges and opportunities for enhancing adolescent nutrition in urban Ethiopia. We observed that many adolescents in Addis Ababa have suboptimal diets and significant gaps in nutrition knowledge, despite generally positive attitudes toward healthy eating. Socio-economic disparities are clear, with adolescents from higher-income (private school) backgrounds showing better DDS and knowledge than their lower-income (public school) peers. These results indicate that current efforts are not enough to ensure all adolescents have access to a healthy diet. Targeted strategies are necessary to bridge knowledge gaps and increase the accessibility and appeal of healthy foods. Improving adolescent nutrition is vital for Ethiopia to reach its national nutrition goals and protect the health of future generations. Our findings also highlight specific areas for action, especially by using schools as key platforms for change. Aligning with global nutrition standards and strengthening national youth-focused programs can help stakeholders make significant progress in reducing adolescent malnutrition. To foster a healthier generation, national nutrition policies should set clear goals for improving dietary diversity and micronutrient intake, promote multisectoral collaboration to combat adolescent malnutrition, and regulate unhealthy food marketing. Schools should incorporate nutrition education at all levels, provide affordable healthy food options, and offer targeted support to public schools in low-income areas. Additional research, particularly longitudinal studies on the long-term effects of adolescent diets and the impact of school-based nutrition programs, is essential.

## Data Availability

The raw data supporting the conclusions of this article will be made available by the authors, without undue reservation.
